# SdiA Improves the Acid Tolerance of *E. coli* by Regulating GadW and GadY Expression

**DOI:** 10.3389/fmicb.2020.01078

**Published:** 2020-06-03

**Authors:** Xingyan Ma, Shebin Zhang, Zhenjie Xu, Honglin Li, Qian Xiao, Feng Qiu, Weizheng Zhang, Yifei Long, Dexiang Zheng, Bin Huang, Cha Chen, Yang Lu

**Affiliations:** ^1^Department of Laboratory Medicine, The Second Affiliated Hospital of Guangzhou University of Chinese Medicine, Guangzhou, China; ^2^Department of Laboratory Medicine, The First Affiliated Hospital of Sun Yat-sen University, Guangzhou, China; ^3^The Second Clinical College, Guangzhou University of Chinese Medicine, Guangzhou, China; ^4^Department of Laboratory Medicine, Nanhai Hospital, Southern Medical University, Foshan, China

**Keywords:** SdiA, glutamate decarboxylase W (GadW), glutamate decarboxylase Y (GadY), acid tolerance, *Escherichia coli*

## Abstract

The acid tolerance mechanism is important for *Escherichia coli* to resist acidic conditions encountered in mammalian host digestive tract environment. Here, we explored how the LuxR protein SdiA influenced *E. coli* acid tolerance ability in the context of the glutamate- and glutamine-dependent acid resistance system (AR2). First, using a growth and acid shock assay under different acid stresses, we demonstrated that the deletion of *sdiA* in *SM10*λ*pir* or *BW25113* led to impaired growth under the acidic environment of pH 3–6, which was restored by complementary expression of SdiA. Next, transcriptome sequencing and qPCR disclosed that the expression of glutamate decarboxylase W (GadW) and GadY, the key members of the AR2 system, were regulated by SdiA. Further, β-galactosidase reporter assays showed that the promoter activity of *gadW* and *gadY* was positively regulated by SdiA. Moreover, qPCR and β-galactosidase reporter assays confirmed that the regulation of SdiA on GadW, but not GadY, could be enhanced by quorum sensing (QS) signal molecules AHLs. Collectively, these data suggest that SdiA plays a crucial role in acid tolerance regulation of *E. coli*. Our findings provide new insights into the important contribution of quorum sensing system AHLs–SdiA to the networks that regulate acid tolerance.

## Introduction

Enteric organisms, such as *Escherichia coli*, often colonize in the host’s gastrointestinal tract and cause disease. During this process, the acidic stress in the host’s stomach sets a barrier to enteric pathogens. *E. coli* has a remarkable ability to withstand low pH environment by activating several acid resistance (AR) systems, especially the glutamate-dependent AR system (AR2), which is the most effective AR system to cope with extreme acid stress (Lund et al., 2014; Zhao et al., 2018). The AR2 system has three structural components including two isoforms of glutamate decarboxylases (GadA and GadB), and the aminobutyrate (GABA) antiporter GadC. Among them, *gadB* and *gadC* genes are co-transcribed. The *gadA* gene is 2.1 Mb from *gadBC* and exists in the acid fitness island (AFI) (De Biase and Pennacchietti, 2012). This AFI comprises 14 genes related to AR. Among these genes, *gadE*, *gadW*, and *gadX* encode regulators of the AR2 system (Ma et al., 2003; Tramonti et al., 2008; Seo et al., 2015). In addition, a small RNA, GadY, is also involved in the regulation of AR2 system by stabilizing the *gadX* mRNA (Opdyke et al., 2004; Tramonti et al., 2008). The GadX–GadY–GadW circuit can strengthen the AR2 system by activating *gadE* transcription and directly binding to the promoter regions of *gadA* and *gadBC* (Tramonti et al., 2006). However, the regulation of this circuit remains unclear.

In adaptation to environmental change, quorum sensing (QS) system plays a significant role through chemical communication between bacterial cells. In Gram-negative bacteria, this communication mainly uses signal molecules, N-acyl homoserine lactones (AHLs), and cognate LuxR transcription factor (Schuster et al., 2013). The AHLs can recognize specific LuxRs by their variable acyl chains and regulate gene transcription (Fuqua and Greenberg, 2002). Some organisms, such as *Escherichia* and *Salmonella*, only have LuxR-type protein SdiA but do not produce AHLs. However, numerous studies have demonstrated that this orphan LuxR-type receptor is able to bind to DNA and regulates gene transcription with or without AHLs, and then involves in interspecies signaling (Yamamoto et al., 2001; Dyszel et al., 2010; Nguyen et al., 2015). Moreover, AHLs can improve the transcriptional regulation ability of SdiA through enhancing SdiA stability and DNA-binding affinity, thus regulating many gene transcription in an SdiA-dependent manner (Van Houdt et al., 2006; Lee et al., 2008; Nguyen et al., 2015). In addition, SdiA of enterohemorrhagic *E. coli* O157:H7 (EHEC) can be constitutively activated by the binding of molecule 1-octanoyl-rac-glycerol (OCL) in the absence of AHLs (Nguyen et al., 2015). As increasing investigations about SdiA have been done, it is generally accepted that SdiA plays a vital role in facing different environments including acid environment (Dyszel et al., 2010; Hughes et al., 2010). Nevertheless, the function of SdiA in AR and its underlying mechanisms remain largely unknown.

In this study, we focused on the role of SdiA in AR and explored how SdiA regulated the Gad system. We first confirmed the link between SdiA and acid tolerance by investigating the effect of the knockout and overexpression of *sdiA* on cell growth in media acidifed by citric acid. Subsequently, the transcriptome, qPCR and β-galactosidase reporter analysis were performed to shed some light on the mechanism. Our works offer a deeper understanding of the role of SdiA in the regulation of AR.

## Materials and Methods

### Bacterial Strains, Plasmids, and Growth Conditions

The bacterial strains and plasmids used in this study are listed in Supplementary Table S1. The construction of *sdiA*-deficient or -overexpression strains has been described in our previous study (Lu et al., 2017). Bacteria were grown in LB broth or on LB agar unless otherwise indicated. When necessary, antibiotics were used at the following concentrations: 100 μg/ml ampicillin (AMP) and 16 μg/ml chloramphenicol (CM).

### Plasmid Construction

The plasmid pQF50 containing a promoter-less *lacZ* reporter was used for *gadY* and *gadW* promoter analysis. The DNA fragments of the *gadY* promoter spanning −245 to +13 and *gadW* promoter spanning −258 to −1 were PCR amplified and inserted into *Bam*HI/*Hind*? sites upstream of the *lacZ* reporter in pQF50 to generate pQF50-P*gadY* and pQF50-P*gadW*, respectively. All constructs were confirmed by direct sequencing. Primers used in this study are listed in Supplementary Table S2.

### RNA Sequencing and Bioinformatics Analysis

The wild-type strain, *sdiA* gene-deficient and -overexpression strains in SM10λpir were grown overnight (8∼10 h) at 37°C, and then 100 μl of the cultures were added into 3 ml of LB to grow to mid-exponential phase (0.5 McFarland standard). Then, total RNA was extracted using RNAprep Pure Cell/Bacteria Kit (Tiangen, Beijing, China), and the entire sequencing was conducted by the BGI Company. Experiments were performed in triplicate. The raw data had been submitted to SRA database of NCBI (accession number: PRJNA627821, SRP258248). The expression of Unigene was calculated by RPKM method (Reads Per kb per Million reads).

### Real-Time PCR

Total RNA was extracted using RNAiso Plus reagent (Takara, Dalian, Liaoning, China). Reverse transcription (1 μg of total RNA) was performed with the PrimeScript RT Reagent Kit (code No. RR047A; Takara, Dalian, Liaoning, China). The cDNA was subjected to qPCR on a ViiA^TM^ 7 Dx system (Applied Biosystems, Foster, CA, United States) using SYBR Green qPCR Master Mix (Takara, Dalian, Liaoning, China). The expression levels of the target genes were normalized to the expression of an internal control gene (*rpoD*) using the 2^–ΔΔCt^ method. The sequences of the primers are listed in Supplementary Table S2.

### Growth Assay

Growth and acid shock assay were performed as described previously, with some modifications (Gao et al., 2018). *E. coli* strains were grown overnight (8∼10 h) in LBG medium (LB medium supplemented with 0.4% glucose) of pH 7.0 at 37°C. Then the bacteria was collected and re-cultured in LBG medium at pH 7.0 or LBG medium acidified by citric acid to pH 6, pH 5, pH 4, and pH 3 for 24 h at 37°C in a 96-well round bottom plate, and the OD_600_ values were determined at the indicated time points. All of the tests were carried out independently at least in triplicate.

### Acid Shock Assay

*Escherichia coli* strains were grown overnight (8∼10 h) in LBG medium of pH 7.0 at 37°C. Then the bacteria was collected and re-cultured in LBG medium at pH 7.0 or LBG medium acidified by citric acid to pH 6, pH 5, pH 4, and pH 3 for 2 h at 37°C in a 96-well round bottom plate. After the acid shock, the cultures were serially diluted, plated on LB agar, incubated at 37°C overnight, and then photographed. All of the tests were carried out independently at least in triplicate.

### β-Galactosidase Assays

β-Galactosidase assays were carried out by the Miller method when cells were grown to mid-log phase at 37°C at pH 7.0 (Giacomini et al., 1992). All of the tests were carried out independently at least in triplicate.

### Treatment With AHLs

The indicated *E. coli* strains were grown overnight in LB medium at 37°C, and then 100 μl of the cultures was added into 3 ml of LB that contains DMSO (control) or C4-HSL and 3-oxo-C12-HSL (40 μM each) and grown for 6 h at 37°C, followed by RT-qPCR or b-galactosidase activity analysis. All of the tests were carried out independently at least in triplicate.

### Statistical Analysis

Data of the results from multiple independent experiments were expressed as the mean ± standard deviation (SM). The differences between groups were analyzed using Student’s *t*-test when two groups were compared or one-way ANOVA when more than two groups were compared. All analyses were performed using GraphPad Prism, version 5 (GraphPad Software, Inc., San Diego, CA, United States). Differences with a value of 0.01 < *P* < 0.05 are represented by ^∗^, *P* < 0.01 are represented by ^∗∗^, and *P* < 0.001 are represented by ^∗∗∗^.

## Results

### SdiA Enhances Acid Tolerance Ability of *E. coli*

**FIGURE 1:**
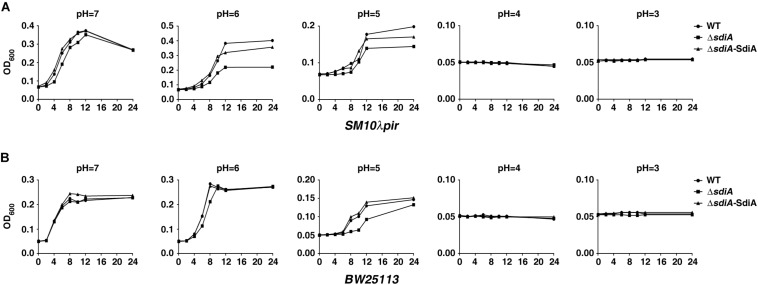
Growth of *sdiA*-deficient and overexpression strains under acid stress. **(A,B)** The indicated *Escherichia coli SM10*λ*pir* and *E. coli BW25113* strains in exponential phase were collected and re-cultured for 24 h in LBG medium with different initial pH values obtained by the addition of citric acid. The samples were collected at the indicated time points, and the OD_600_ values were determined. The curve of each strain growth was shown. WT, *E. coli SM10*λ*pir* or *E. coli BW25113* carrying pROp200; Δ*sdiA*, *E. coli SM10*λ*pir*Δ*sdiA* or *E. coli BW25113*Δ*sdiA* carrying pROp200; Δ*sdiA*-SidA, *E. coli SM10*λ*pir*Δ*sdiA* or *E. coli BW25113*Δ*sdiA* carrying pROp200-*sdiA*.

To assess the influence of SdiA on acid tolerance of *E. coli*, a series of growth assays under different acid stress (24 h of incubation in LBG medium acidified to pH 3–7 by citric acid) was performed in *sdiA* gene-deficient strain of *SM10*λ*pir* and *BW25113* (Δ*sdiA*), as well as *sdiA*-overexpression strain in Δ*sdiA* mutation (Δ*sdiA/*SdiA). For *SM10*λ*pir*, when the pH was down to 6 or 5, deficiency of *sdiA* impaired the growth through the whole process, compared to the wild-type strain, which was restored by introduction of *sdiA*-overexpression plasmid (Figure 1A). For *BW25113*, compared to *sdiA*-deficient strain, wild-type and *sdiA*-overexpression strain also showed growth advantage in the early stage under pH 6 and all the stage under pH 5 (Figure 1B). For both *SM10*λ*pir* and *BW25113*, when the pH was down to 4 or 3, the OD_600_ value of wild-type and modified strains showed no difference, as a result of no growth (Figures 1A,B).

**FIGURE 2:**
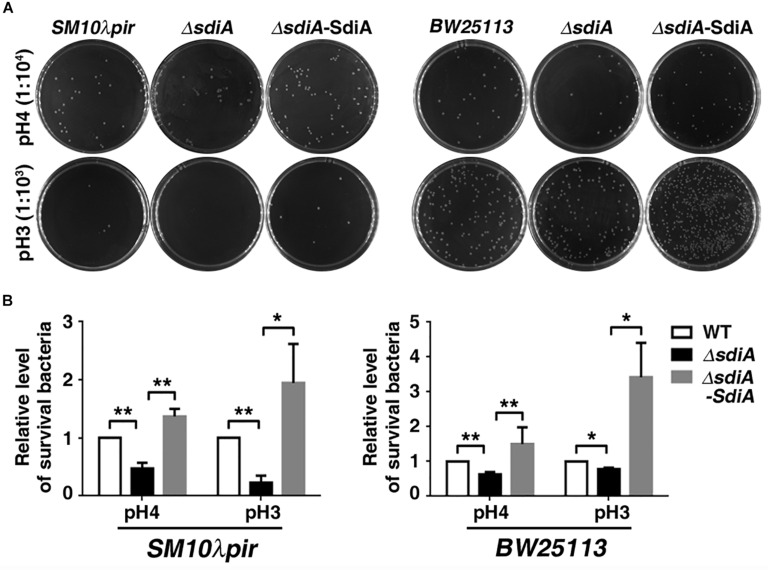
Survival of *sdiA*-deficient and overexpression strains after acid shock. **(A,B)** *E. coli SM10*λ*pir* and *E. coli BW25113* wild-type strain, *sdiA*-deficiency strain (*E. coli SM10*λ*pir*Δ*sdiA*, *E. coli BW25113*Δ*sdiA*), *sdiA*-deficiency and overexpression strain (*E. coli SM10*λ*pir*Δ*sdiA*-SdiA, *E. coli BW25113*Δ*sdiA*-SdiA) (2.0 × 10^5^) were incubated for 2 h in LBG medium acidified by citric acid. The images represent 1:10^4^ (pH 4) or 1:10^3^ (pH 3) of the serial dilutions of the cultures in 10-fold steps. *P < 0.05; **P < 0.01.

An acid shock assay (2 h of incubation in LBG medium acidified to pH 3–7 by citric acid) was further carried out to investigate the effect of SdiA on acid tolerance under extreme acid stress. The result showed that deficiency of *sdiA* resulted in decreased survival of both *SM10*λ*pir* and *BW25113* under the acid stress of pH 4 or 3, whereas overexpression of SdiA abrogated this effect (Figures 2A,B).

Collectively, these data suggest that SdiA plays a crucial role in acid tolerance ability of *E. coli*.

### SdiA Promotes the Expression of GadW and GadY

Next, we explored the molecular mechanisms responsible for the function of SdiA that were observed above. The transcriptomes of *SM10*λ*pir*, *SM10*λ*pir*Δ*sdiA*, and *SM10*λ*pir*Δ*sdiA*-SdiA were determined, and we mainly focused on AR2 system genes. The results showed that the transcription of *gadA*, *gadB*, *gadC*, *gadE*, and *gadX* was hardly changed in *sdiA-*knockouted strain, while overexpression of SdiA enhanced *gadA*, *gadB*, and *gadC* expression. This is consistent with the observation that many genes of *E. coli* that respond to plasmid-based expression of SdiA are largely more different than those that respond to chromosomal SdiA (Dyszel et al., 2010). Noteworthy, the expression of GadW and GadY was changed in both *sdiA* gene deficient and its compensatory strains, with the down-regulation in *SM10*λ*pir*Δ*sdiA*, and up-regulation upon SdiA overexpression (Table 1).

**TABLE 1:** Transcript level of AR2 genes in *SM10*λ*pir sdiA* deficient and complemented strains.

	**WT**	**Δ*sdiA***	**Δ*sdiA*-SdiA**
*sdiA*	448	0	11416
*gadA*	327.5	262.23	462.62
*gadB*	58.5	56.77	138.38
*gadC*	93	61	159
*gadE*	449	360	470
*gadW*	387	147	372
*gadX*	751	563	791
*gadY*	14	8	17

*The calculation of Unigene expression uses RPKM method (reads per kb per million reads).*

**FIGURE 3:**
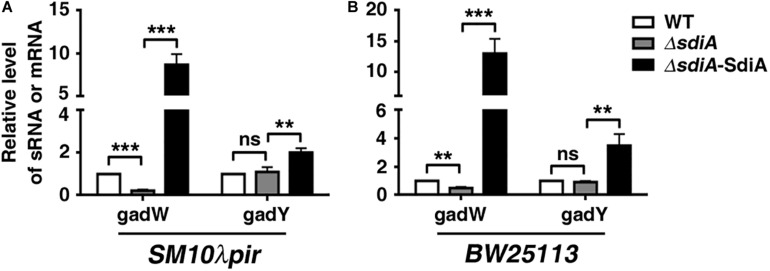
SdiA promotes glutamate decarboxylase W (GadW) and GadY expression. **(A)** The influence of SdiA on GadW and GadY expression in *E. coli SM10*λ*pir*. **(B)** The influence of SdiA on GadW and GadY expression in *E. coli BW25113*. *E. coli SM10*λ*pir* or *E. coli BW25113* (WT) and the *sdiA-*deficient strains (Δ*sdiA*) carrying pROp200, *E. coli SM10*λ*pir*Δ*sdiA* carrying pROp200-SdiA (Δ*sdiA*-SdiA) were cultured in LB for 6 h, followed by real-time PCR analysis; the *rpoD* gene was used as an internal control. **P < 0.01; ***P < 0.001.

Based on the obvious change in transcriptome data, GadW and GadY were chosen for further analysis by quantitative RT-PCR. Conformably, deficiency of *sdiA* in both *SM10*λ*pir* and *BW25113* resulted in downregulation of GadW, which was reversed by restoration of *sdiA*. For GadY, the expression was barely changed in *sdiA*-deficient strain, but elevated in *sdiA*-overexpression strain (Figure 3). Taken together, these results implicate that SdiA may positively regulate GadW and GadY expression.

### SdiA Regulates Transcription Activity of GadW and GadY

**FIGURE 4:**
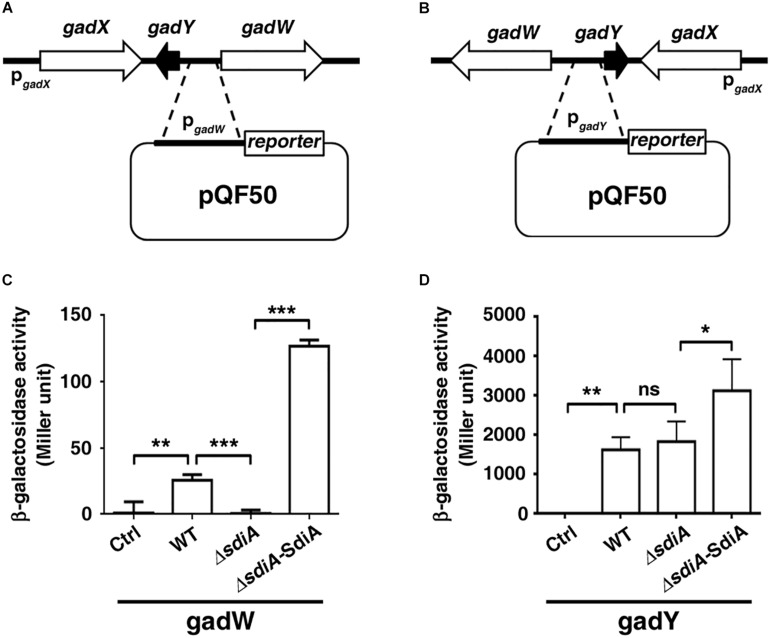
The promoter activity of *gadW* and *gadY* is regulated by SdiA. **(A,B)** Diagram of the *gadW* and *gadY* promoter and principles of the *in vivo* investigation of their activity. **(C,D)** The influence of SdiA on the promoter activity of *gadW* and *gadY*. The *E. coli BW25113* strains carrying the reporter pQF50, pQF50-P*gadW*, or pQF50-P*gadY* combined with pROp200 or pROp200-SdiA were grown to mid-log phase, subjected to β-galactosidase activity assay. Values are mean ± SD of at least three independent experiments. *P < 0.05; **P < 0.01; ***P < 0.001.

We subsequently evaluated the influence of SdiA on the transcription activity of the *gadW* and *gadY* promoter regions. The DNA fragment of *gadW* or *gadY* promoter was cloned upstream of the β-galactosidase gene in the pQF50-promoter reporter, respectively (Figures 4A,B). When transformed into *BW25113* (without endogenous β-galactosidase), the β-galactosidase activity of pQF50-P*gadW* and pQF50-P*gadY* was greatly elevated, compared to that of the control. More importantly, deficiency of *sdiA* impaired the activity of pQF50-P*gadW* but hardly affected that of pQF50-P*gadY*, while overexpression of SdiA enhanced both promoter activity of *gadW* and *gadY* (Figures 4C,D). These observations were consistent with the above quantitative RT-PCR results.

**FIGURE 5:**
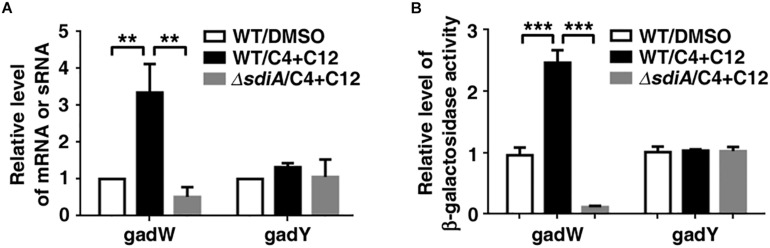
The effect of N-acyl homoserine lactones (AHLs) on the expression of *gadW* and *gadY*. **(A)** AHLs promoted *gadW* but not *gadY* expression. *E. coli SM10*λ*pir* was cultured in the presence of DMSO or 40 μM C4-HSL and 3-oxo-C12-HSL for 6 h, followed by real-time PCR analysis. **(B)** AHLs enhanced the promoter activity of *gadW* but not *gadY*. *E. coli BW25113* was cultured in the presence of DMSO (Ctrl) or 40 μM C4-HSL and 3-oxo-C12-HSL for 6 h, followed by b-galactosidase activity analysis. Values are mean ± SD of at least three independent experiments. **P < 0.01; ***P < 0.001.

Moreover, it has been shown that transcriptional regulatory function of SdiA may be increased by AHLs. We further checked the influence of AHLs on the promoter activity and expression level of *gadW* and *gadY*. As shown in Figures 5A,B, treatment with 3-oxo-C12-HSL and C4-HSL not only enhanced the promoter activity of *gadW* but also promoted its expression, which were attenuated by deficiency of *sdiA*. However, this processing seemed to have no effect on the promoter activity and expression of GadY.

Taken together, our results implicate that SdiA may promote acid tolerance of *E. coli* at least partly through regulating GadW and GadY expression.

## Discussion

In this study, we disclosed the role of SdiA in acid tolerance of *E. coli*. Our findings highlight the transcription control of SdiA on GadW and GadY, and provide new insights into the regulatory network of the acid resistance system AR2.

Previous studies have identified AFI as the key factor that improves the tolerance of *E. coli* strains to environmental acidic stress (Foster, 2004). One of the essential AFI regulators, GadE, was a LuxR-like protein. In the present study, we focused on another LuxR protein SdiA. Most recent publications about SdiA have reported its gene regulatory function (Dyszel et al., 2010; Nguyen et al., 2015; Sabag-Daigle et al., 2015). A few reports revealed the roles of SdiA in diverse biological processes (Smith et al., 2011), such as cell division (Sitnikov et al., 1996), antibiotic resistance (Yang et al., 2006; Tavío et al., 2010), virulence (Kanamaru et al., 2000), and biofilm formation (Culler et al., 2018). Here, gain- and loss-of-function studies showed that SdiA positively regulated mild and extreme acid (pH 3–6) tolerance ability of both *E. coli* strains *SM10*λ*pir* and *BW25113*. Since acid stress can damage bacteria cells and impair their growth, and SdiA was activated during the transit of *Salmonella* through turtles (Smith et al., 2008), we conferred that SdiA might favor *E. coli* to resist acidic conditions encountered during transit through the mammalian host gastric environment and prolonged exposure to the mildly acidic environment of the host gut.

To date, many SdiA regulon members have been described (Dyszel et al., 2010; Abed et al., 2014; Shimada et al., 2014; Sabag-Daigle et al., 2015). Here, we report the identification of SdiA-regulated genes *gadW* and *gadY* through transcriptome sequencing and qPCR. Although the observation that the *gad* system can be activated by SdiA even in the absence of AHLs has been previously reported (Dyszel et al., 2010; Hughes et al., 2010), how SdiA directly activates transcription of the *gad* system is unclear. It seems that genes with specific DNA sequences (SdiA-box) 5′-AAAAG(N8)GAAAA-3′ in the promoter region may be the potential targets of SdiA (Yamamoto et al., 2001). Our bioinformatics analysis discovered a DNA motif 5′-AAAAT(N18)TAAAA-3′ and 5′-AAAAC(N18)CAAAA-3′ in the *gadY*–*gadW* intergenic region. Further, β-galactosidase activity test showed that SidA regulated the promoter activity containing the above-mentioned DNA motifs. However, the interaction between SdiA and these sites needed further validation. In addition, the β-galactosidase reporter system showed that the transcription activity of *gadY* arising from *gadY*–*gadW* intergenic region was much higher than that of *gadW* (Figure 4). Nonetheless, the influence of SdiA on the promoter activity of *gadW* was more remarkable than that of *gadY*, indicating that there might be other transcription factors that regulated *gadY* expression.

It has been shown that SdiA is already in a DNA-binding conformation in the absence of AHLs. AHLs enhance this protein’s affinity to DNA, allowing it to regulate transcription of genes that have lower-affinity sites to this protein, whose SdiA regulation occurs only in the presence of AHLs (Nguyen et al., 2015). This was consistent with our observation that SdiA alone can regulate GadW expression, while the presence of C4- and 3-oxo-C12 HSLs enhanced this effect. C4- and 3-oxo-C12 HSLs are the well-known AHLs from *Pseudomonas aeruginosa*. We have previously found that C4- and 3-oxo-C12 HSLs produced by *P. aeruginosa* can inhibit *traI* expression by activating *E. coli* SdiA. However, neither C4- nor 3-oxo-C12 is the most potent ligands for SdiA, thus the response of SdiA to these two AHLs in this system can possibly represent an artifact. For GadY, the expression was barely changed in chromosomal *sdiA*-deficient strain, but elevated in *sdiA*-overexpression strain, which might be explained by the use of high copy number plasmids in this study, and it has been well established that overexpression of SdiA can bypass their requirement for AHL (Michael et al., 2001; Dyszel et al., 2010). More importantly, the presence of AHLs has been suggested in bovine rumen and human gut, and EHEC senses rumen AHLs through SdiA to repress Lee expression and activate the *gad* AR system, which was necessary for efficient EHEC colonization of cattle fed a forage or grain diet (Sperandio, 2010; Sheng et al., 2013; Landman et al., 2018). Future studies are needed to illuminate the role of AHL-SdiA signaling in acid tolerance within the human gastrointestinal tract.

## Data Availability Statement

All data sets generated for this study are included in the article/Supplementary Material.

## Author Contributions

XM, SZ, and ZX performed the majority of the experiments. HL, QX, WZ, DZ, YFL, and FQ also conducted the experiments. XM, BH, CC, and YL participated in the experimental design and data analysis. CC and YL conceived the project, designed and under took the experiments, interpreted data, and wrote the manuscript. All authors read and approved the manuscript.

## Conflict of Interest

The authors declare that the research was conducted in the absence of any commercial or financial relationships that could be construed as a potential conflict of interest.
