# Supplementary material for: SdiA Improves the Acid Tolerance of E. coli by Regulating GadW and GadY Expression
Source: Front Microbiol. 2020 Jun 3;11:1078. doi: 10.3389/fmicb.2020.01078 (PMC7286202; doi:10.3389/fmicb.2020.01078)
Supplement: Supplementary file 1 [file Table_1.docx]

**Table S1. Bacterial strains and plasmids.**

| **Strains** | **Genotype or characteristics** | **Source** |
| --- | --- | --- |
| ***Escherichia coli*** | | |
| *DH5α* | wild-type strain，supE44△lacU169（φ80lacZ△M15）hsdR17 recAl endAl gyrA19 thi-1 relAl, Cm^S^，Amp^S^ | Our lab |
| *BW25113* | wild-type strain，lacIq, DlacZWJ16, DaraBADAH33, DrhaBADLD78, hsdR514 and rrnBT14, Cm^S^，Amp^S^ | Our lab |
| *BW25113**ΔsdiA* | mutants of *E. coli* *BW25113* deficient in *sdiA* gene, Cm^S^，Amp^S^ | Our lab |
| *SM10λpir* | wild-type strain，thi thrleu tonA lacY supE recA::RP4-2-Tc::Mu Km λpir, Cm^S^，Amp^S^ | Our lab |
| *SM10λpirΔsdiA* | mutants of *SM10λpir* deficient in *sdiA* gene， Cm^S^，Amp^S^ | Our lab |
| ***Plasmids*** | | |
| pQF50 | Promter-less *la*cZ reporter plasmid，Amp^R^ | Our lab |
| pQF50-P*gadY* | pQF50 derivative, *la*cZ reporter plasmid，controlled by the constitutive *gadY*promoter，Amp^R^ | This work |
| pQF50-P*gadW* | pQF50 derivative, *la*cZ reporter plasmid，controlled by the constitutive *gadW*promoter，Amp^R^ | This work |
| pSTV28 | Control plasmid ；containing P*_lac_*promoter，Cm^R^ | Our lab |
| pSTV28-*sdiA* | pSTV28 derivative，*Escherichia colisdiA*overexpression plasmid，controlled by the constitutive P*_lac_*promoter，Cm^R^ | Our lab |

Cm^R^and Amp^R^ stand for chloramphenicol and ampicillin resistance, respectively.
