# Supplementary material for: SdiA Improves the Acid Tolerance of E. coli by Regulating GadW and GadY Expression
Source: Front Microbiol. 2020 Jun 3;11:1078. doi: 10.3389/fmicb.2020.01078 (PMC7286202; doi:10.3389/fmicb.2020.01078)
Supplement: Supplementary file 2 [file Table_2.docx]

**Table S2.Sequences of RNA and DNA oligonucleotides**

| Number | Primers | Primer sequence^*^ (5’→3’) | Use for |
| --- | --- | --- | --- |
| **Primers for gene cloning** | | | |
| 1 | Promoter-*gadY*-F | CGC*GGATCC*GATTATCCCTTATATTTCAT | pQF50-P*gady* |
| 2 | Promoter-*gadY*-R | CCC*AAGCTT*TTGTGCTCTCAGTAAGTTAA |  |
| 3 | Promoter-*gadW*-F | CGC*GGATCC* TTGTGCTCTCAGTAAGTTAA | pQF50-P*gadW* |
| 4 | Promoter-*gadW*-R | CCC*AAGCTT* GATTATCCCTTATATTTCAT |  |
| **Primers for qPCR** | | | |
| 5 | RT-*rpoD*-F | GGGATCAACCAGGTTCAATG | qRT-PCR |
| 6 | RT-*rpoD*-R | GGTGCCAGATCTTCTTCTGC |  |
| 7 | RT-*gadY*-F | CAAAGTTTCCCGTGCCAA | qRT-PCR |
| 8 | RT-*gadY*-R | CATAGGGGACCGGGAAGA |  |
| 9 | RT-*gadW*-F | TTGCGTGGTAGCTGACGAAT | qRT-PCR |
| 10 | RT-*gadW*-R | TTTGTTCACCGGATACGCGA |  |

^*^ The underlined sequences denote restriction enzyme site.
